# Supplementary material for: Characterising nanobody developability to improve therapeutic design using the Therapeutic Nanobody Profiler
Source: Commun Biol. 2026 Jan 31;9:344. doi: 10.1038/s42003-026-09594-y (PMC12963540; doi:10.1038/s42003-026-09594-y)
Supplement: Supplementary file 1 — Supplementary Information [file 42003_2026_9594_MOESM1_ESM.pdf]

Supplementary Information

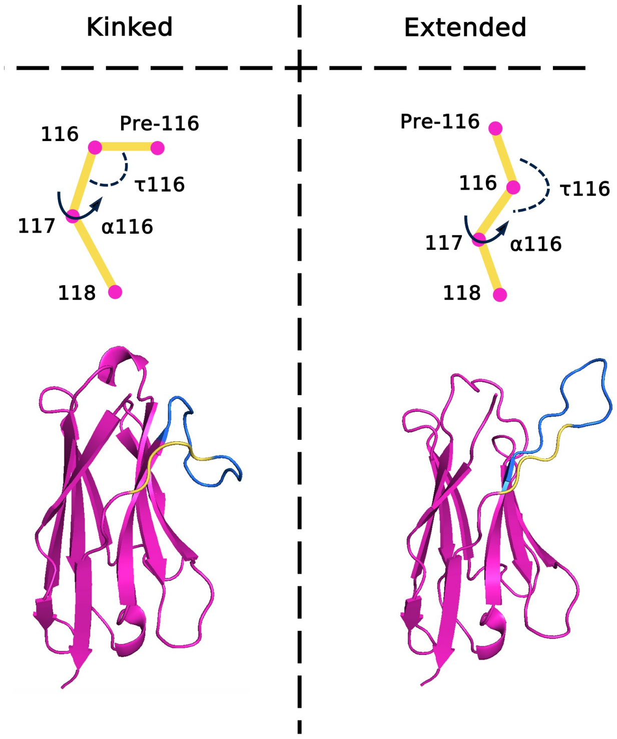

Figure S1. CDR3 conformations can be classified as either 'kinked' or 'extended', following methods in Weitzner *et al.* (2015) and Bahrami-Dizicheh *et al.* (2023) [1, 2]. This classification is based on the τ pseudo-dihedral and α angles between backbone Cα atoms in the CDR3 at positions 116, 117 and 118 (by IMGT numbering). Kinked loops have α and τ angles in the range: $0° < \alpha 116 < 120°$, $85° < \tau 116 < 130°$. Extended loops have α and τ angles in the range: $-100° > \alpha 116$, $100° < \tau 116 < 145°$. Figure is adapted from Bahrami-Dizicheh *et al.* [2].

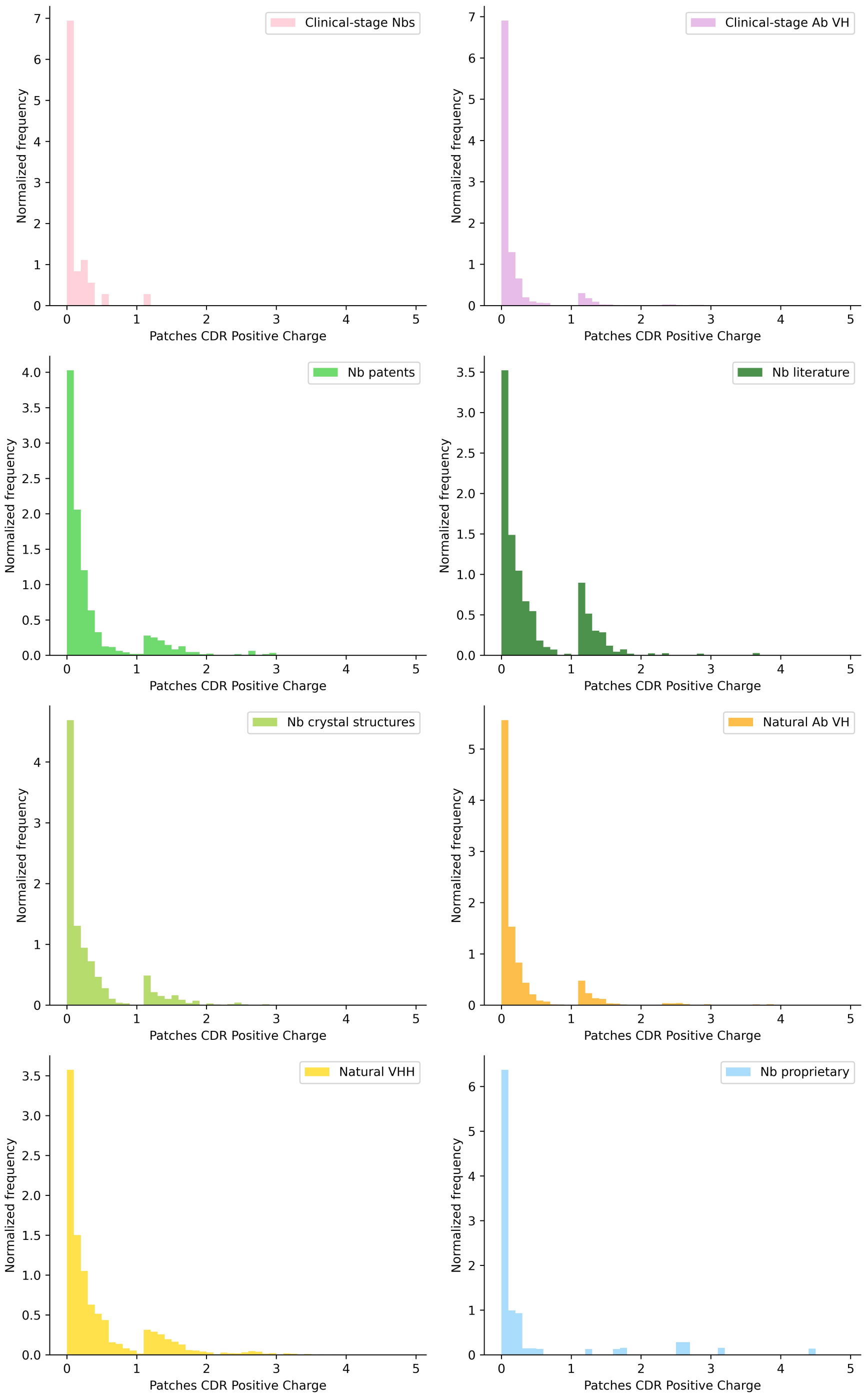

Figure S2. Distributions of scores for patches of positive charge across all our datasets, determined by sampling with replacement all datasets (aside from the 36 clinical-stage nanobodies) with sample size of 36 and 300 samples.

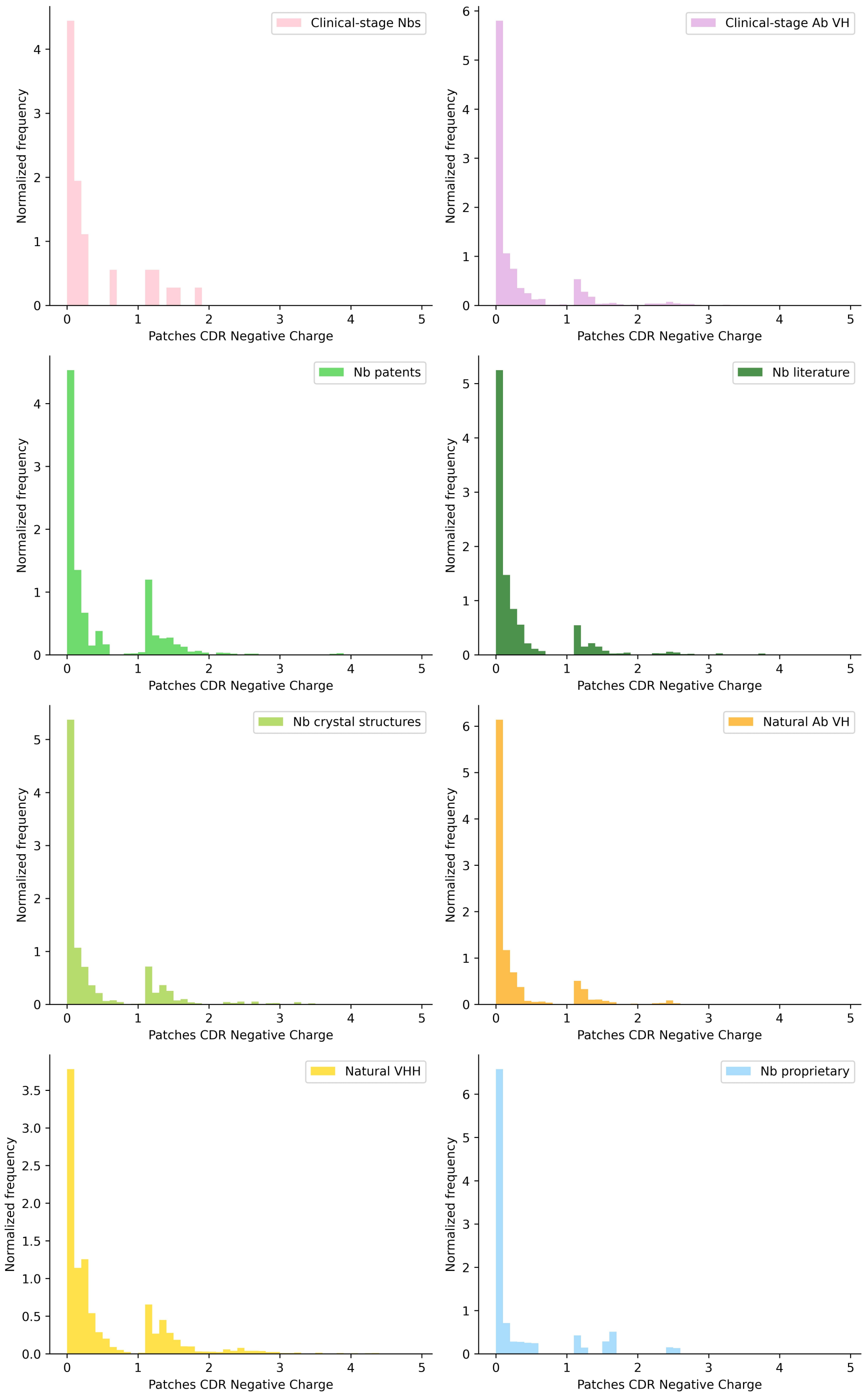

Figure S3. Distributions of scores for patches of negative charge across all our datasets, determined by sampling with replacement all datasets (aside from the 36 clinical-stage nanobodies) with sample size of 36 and 300 samples.

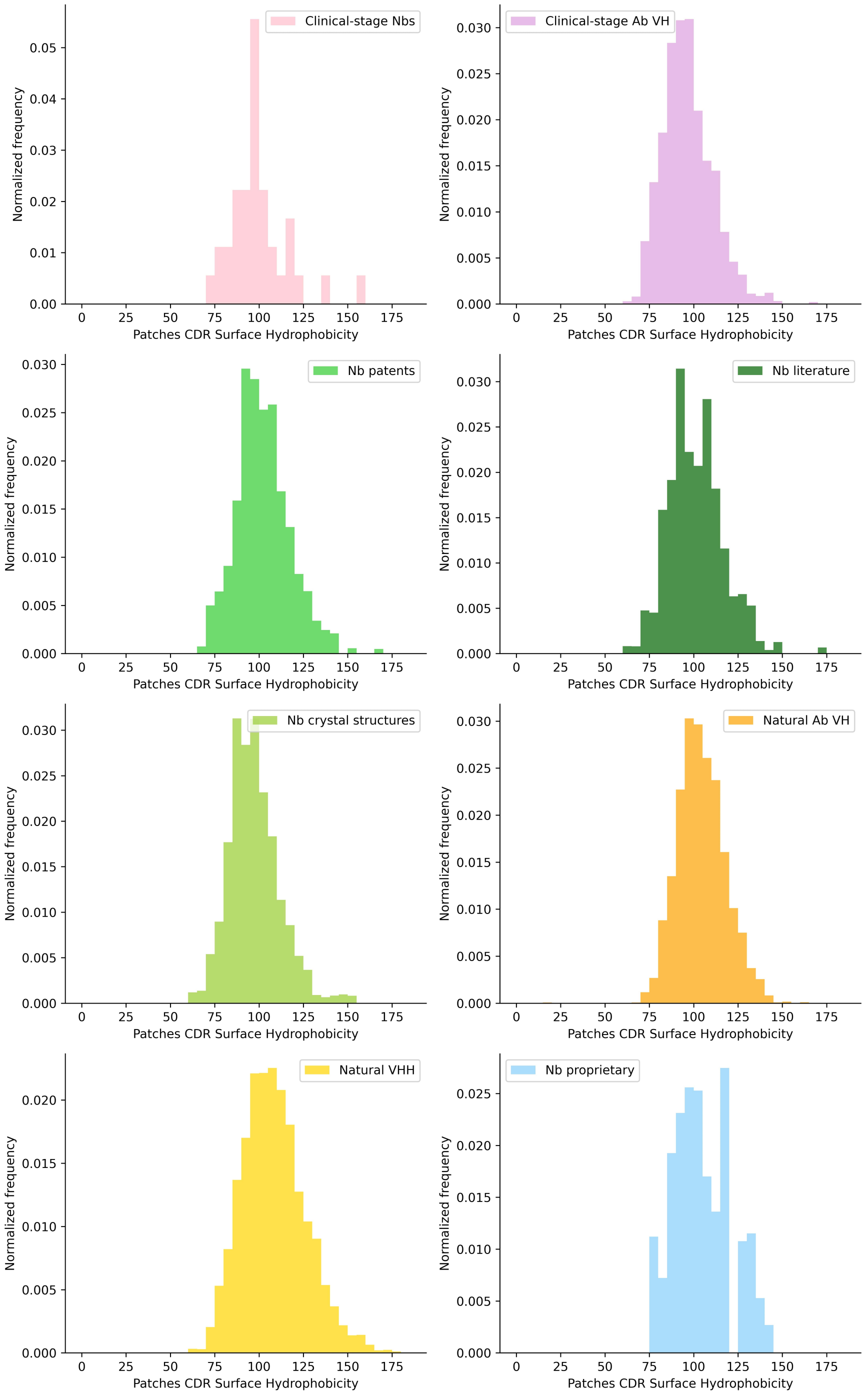

Figure S4. Distributions of scores for patches of surface hydrophobicity across all our datasets, determined by sampling with replacement all datasets (aside from the 36 clinical-stage nanobodies) with sample size of 36 and 300 samples.

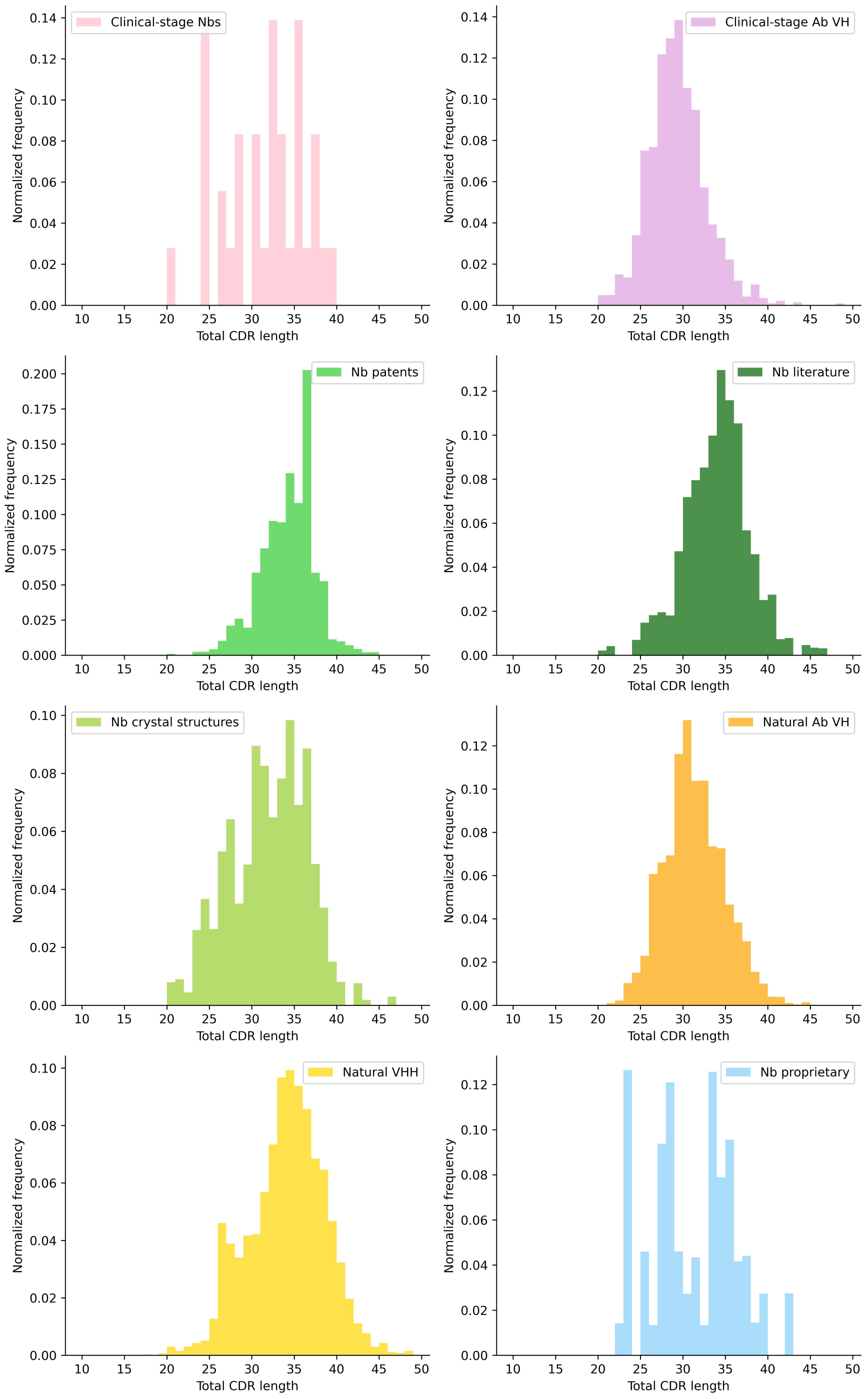

Figure S5. Distributions of total CDR length across all our datasets, determined by sampling with replacement all datasets (aside from the 36 clinical-stage nanobodies) with sample size of 36 and 300 samples.

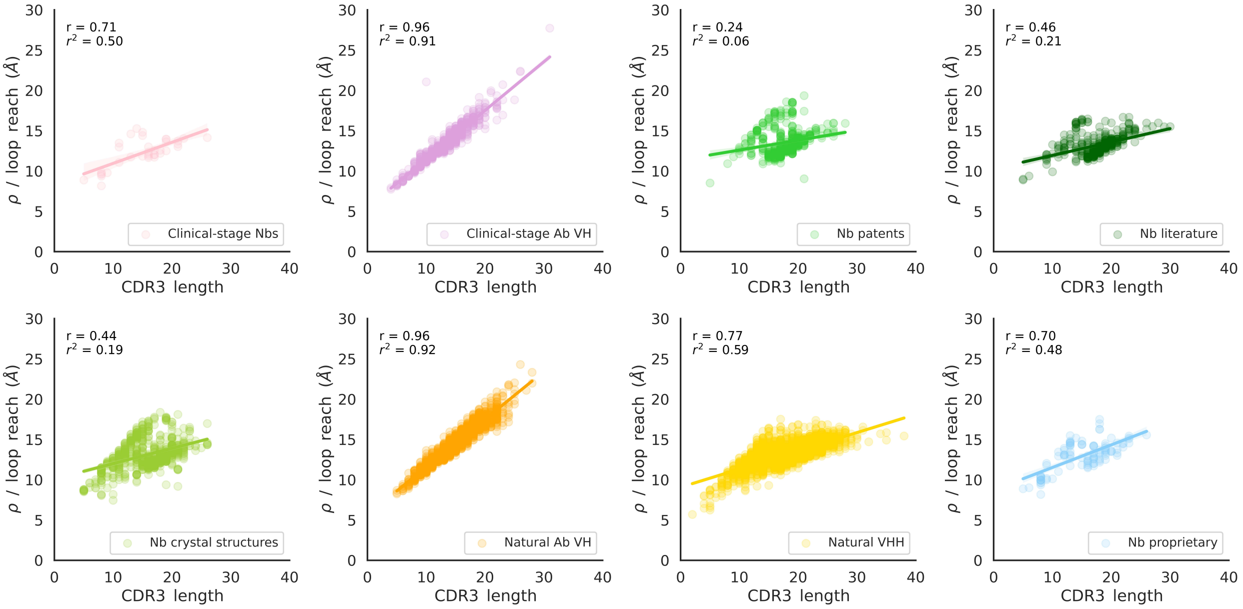

Figure S6. Correlations between reach and length of the CDR3 loop for the different datasets analysed in this study show divergent trajectories for nanobody data, where an increase in length does not translate to greater reach. This relationship can be quantified by loop compactness.

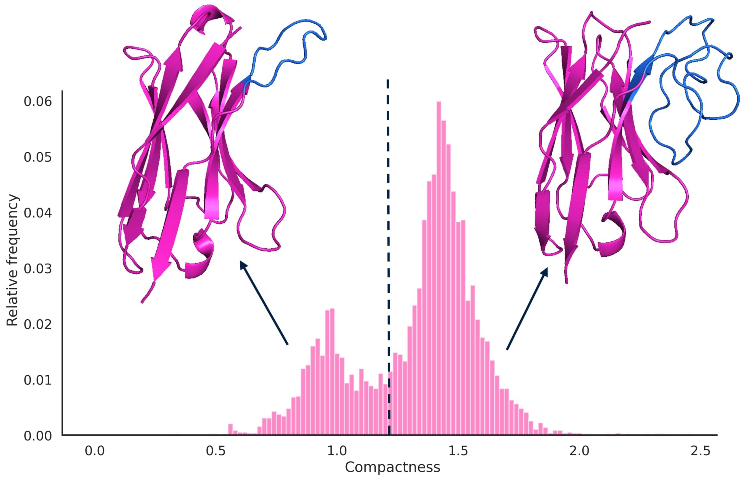

Figure S7. Loop compactness provides a continuous descriptor for CDR3 conformation. The two peaks in the bimodal distribution of compactness for all of our nanobody datasets agree with previous classifications of loop conformations as either extended (lower compactness) or kinked (higher compactness).

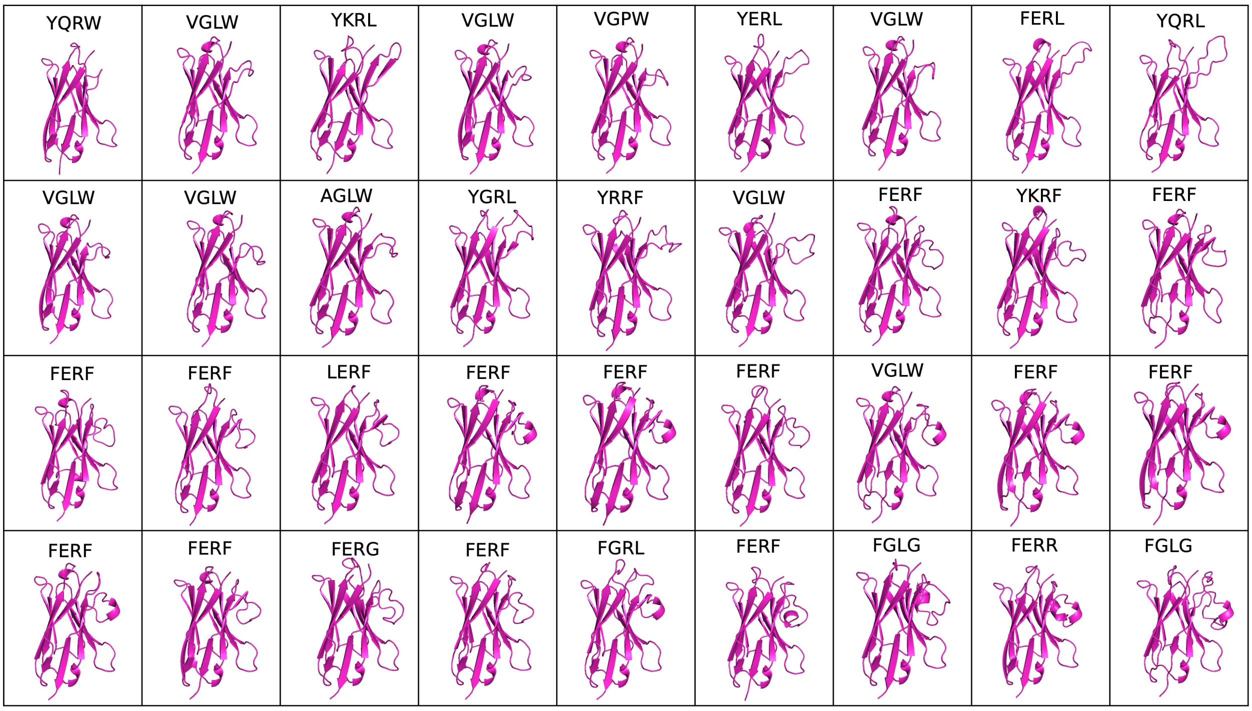

Figure S8. Predicted structures for 36 clinical-stage nanobody sequences in order of increasing compactness (top to bottom, left to right) alongside nanobody tetrad motifs (amino acid identities at IMGT positions 42, 49, 50, 52). As compactness increases, so does CDR3 length and conformational complexity.

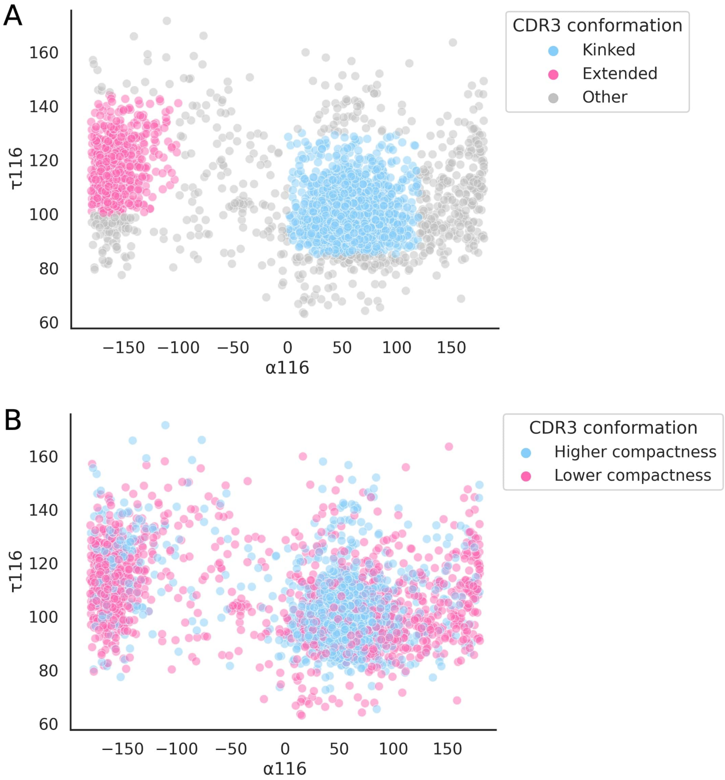

Figure S9. CDR3 conformations can be classified as kinked or extended according to the alpha and tau angles between backbone atoms around position 116 (IMGT numbering) in the CDR3 loop [1, 2]. (**A**) Classification of the CDR3 conformation for all nanobodies from our datasets combined shows that many do not fall into either category using this method. (**B**) Alternatively the CDR3 conformation may be described using compactness. Using the same method to cluster the nanobodies on CDR3 angles, divided into groups of higher and lower compactness, shows the limitations of the classification method given the large conformational diversity.

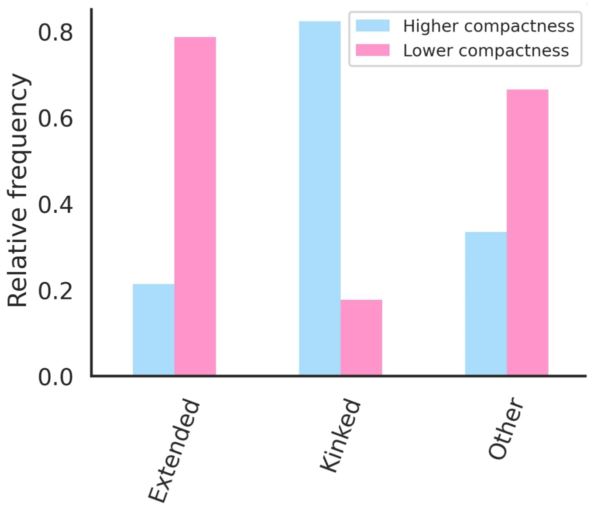

Figure S10. To assess the agreement between compactness and the kinked-extended classifications, a threshold was drawn between the two peaks of the bimodal distribution (**Figure S7**) at 1.25 to separate the data into loops of lower versus higher compactness. Loops with lower compactness closely align with those classified as extended, while those with higher compactness correspond to kinked loops, showing consistency between the compactness measure and the classification method in describing CDR3 conformation.

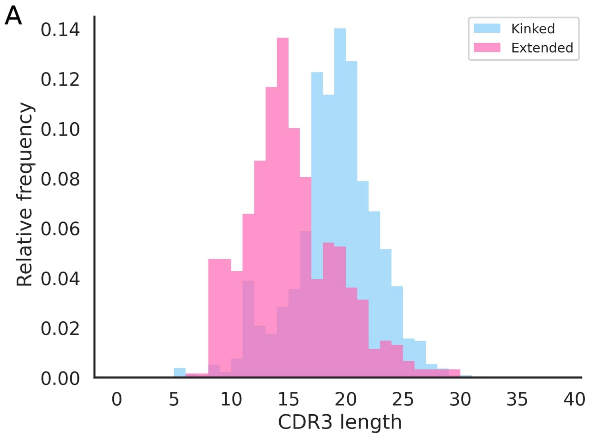

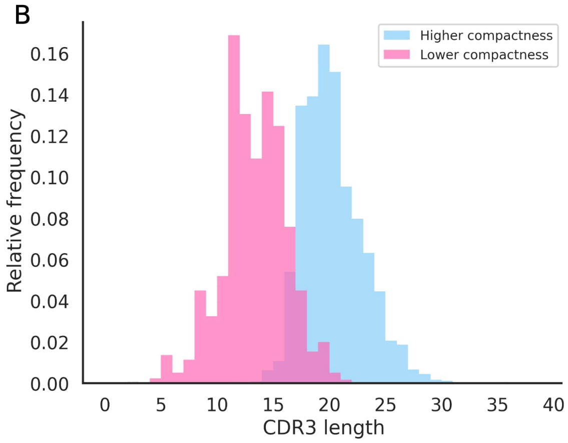

Figure S11. The bimodal distribution for compactness can be used to separate nanobodies into two structural subtypes. We find that these demonstrate distinguishable profiles of secondary features associated with the CDR3 loop structure which are consistent across descriptors for CDR3 conformation. When comparing CDR3 loop length, (**A**) CDR3 loops with a kinked conformation tend to be longer in terms of number of residues than extended conformations. (**B**) CDR3 loops with a higher compactness score tend to be longer than loops of lower compactness.

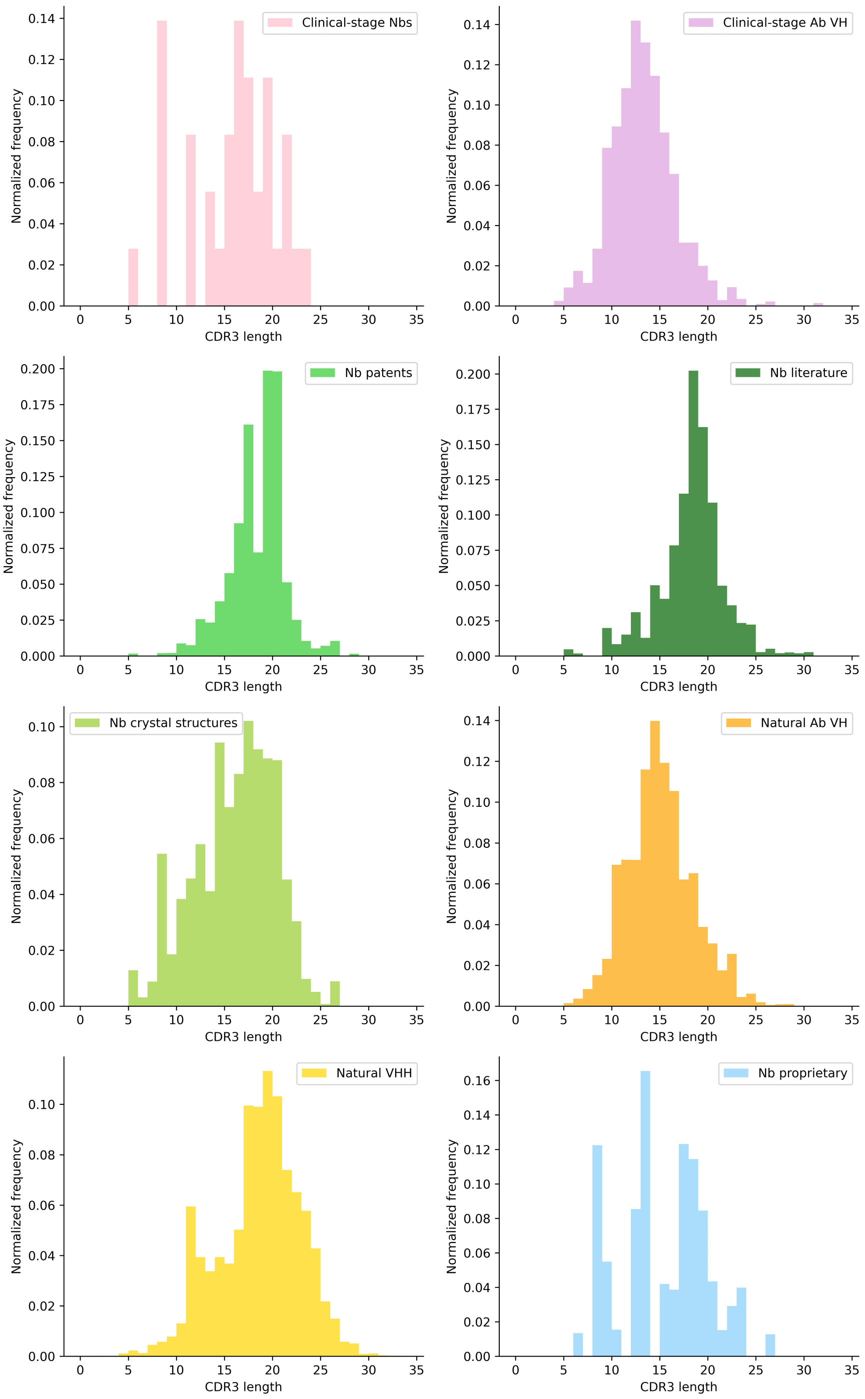

Figure S12. Distributions of CDR3 length across all our datasets, determined by sampling with replacement all datasets (aside from the 36 clinical-stage nanobodies) with sample size of 36 and 300 samples.

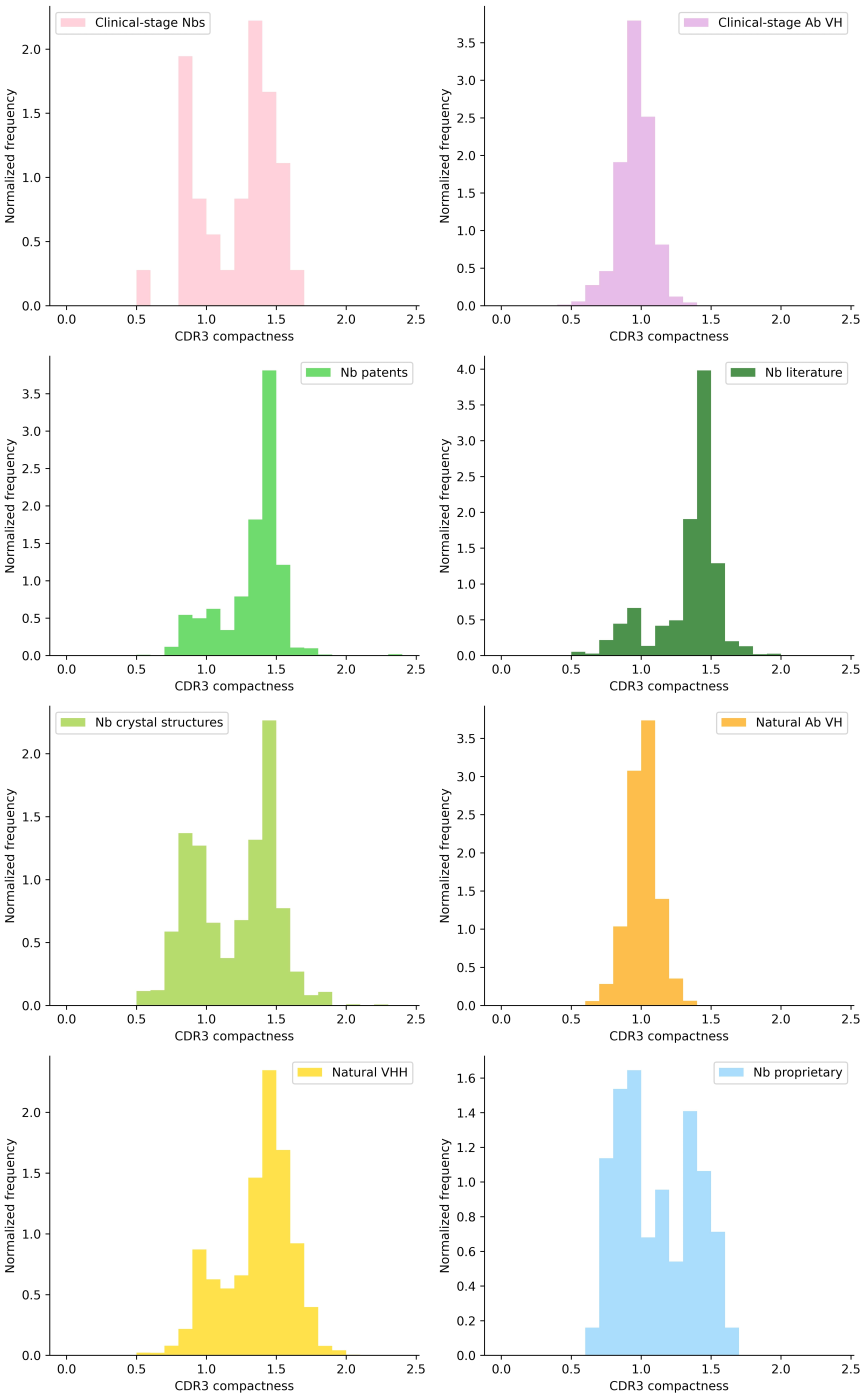

Figure S13. Distributions of CDR3 compactness scores across all our datasets, determined by sampling with replacement all datasets (aside from the 36 clinical-stage nanobodies) with sample size of 36 and 300 samples.

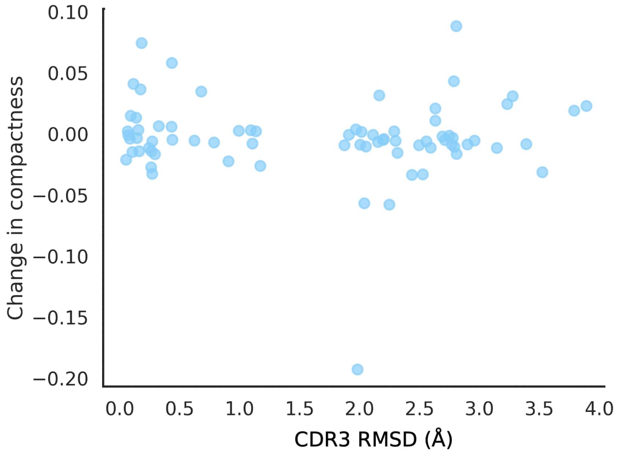

Figure S14. Change in compactness is minimal between bound and unbound pairs of nanobody CDR3 loop structures, and does not show a positive correlation with the RMSD over the bound and unbound CDR3 loops.

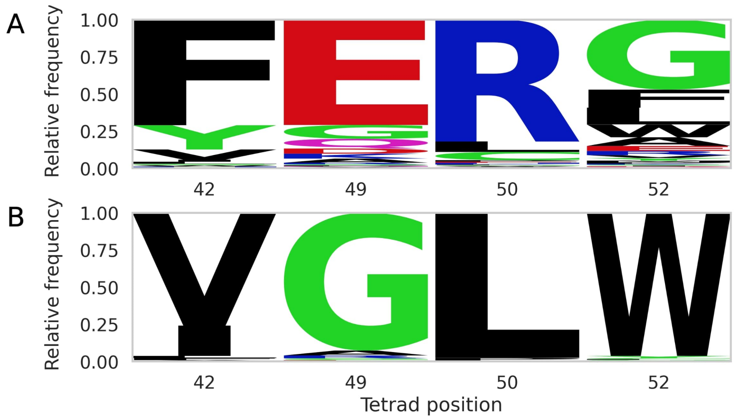

Figure S15. Proportions of amino acids across nanobody tetrad (positions 42, 49, 50 and 52 by IMGT numbering) in our (**A**) nanobody versus (**B**) antibody VH datasets shows that FERG is the most common nanobody motif, whilst VGLW is the most common in antibodies.

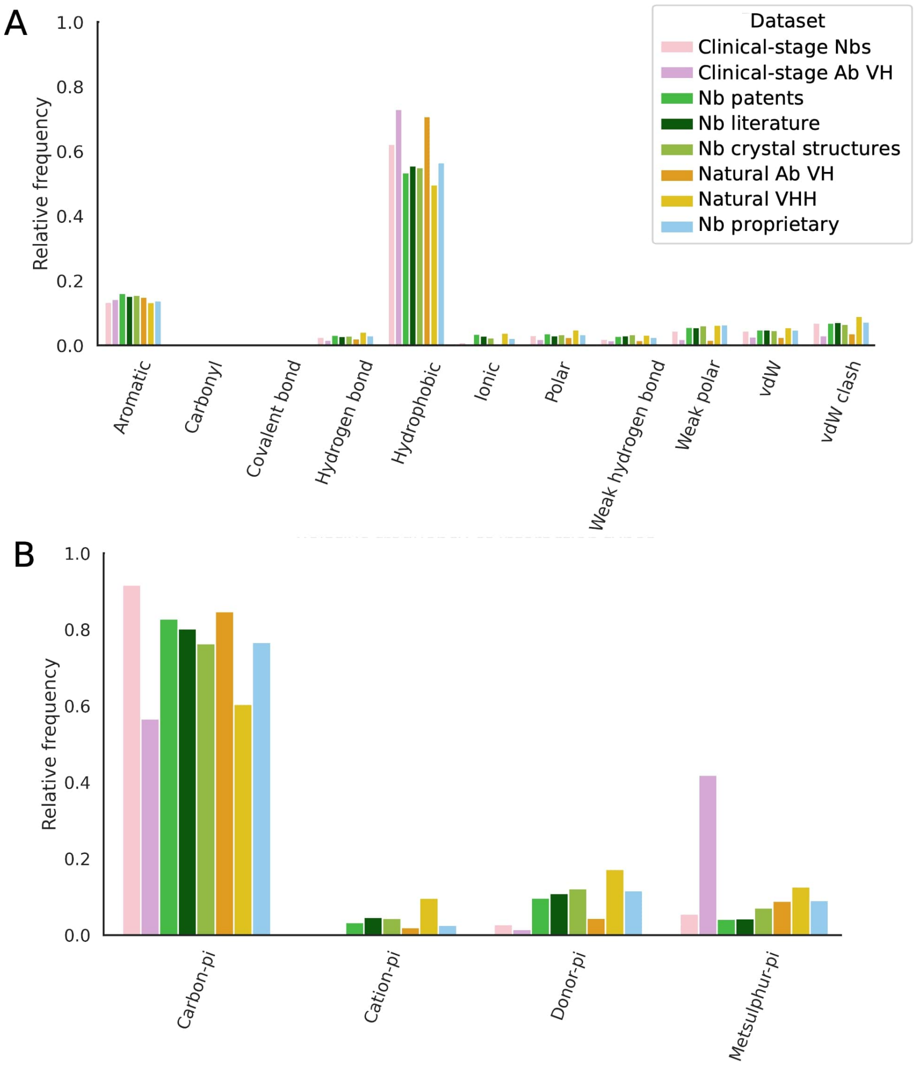

Figure S16. Of pairs of residues in the CDR3 loop and the tetrad positions across all nanobody datasets that were found to interact, for (**A**) atom-atom interactions, hydrophobic, and aromatic interactions were most common. For (**B**) atom-plane interactions, carbon-π interactions were most common.

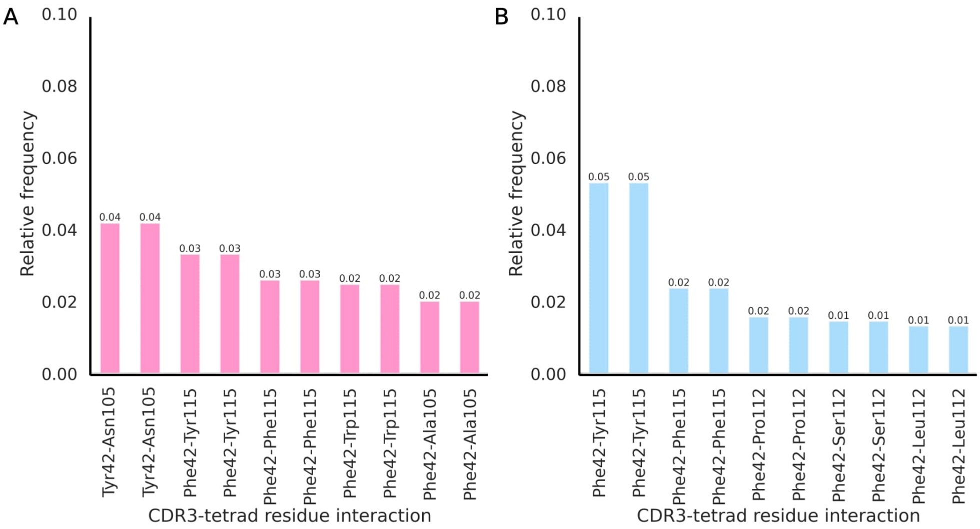

Figure S17. Top 10 most common interacting CDR3-tetrad residue pairs in decreasing order of relative frequency across all nanobody datasets for (**A**) those with CDR3 loops of lower compactness (that tend to extend out from the framework) and (**B**) loops of higher compactness (that tend to fold over the FR2 region).

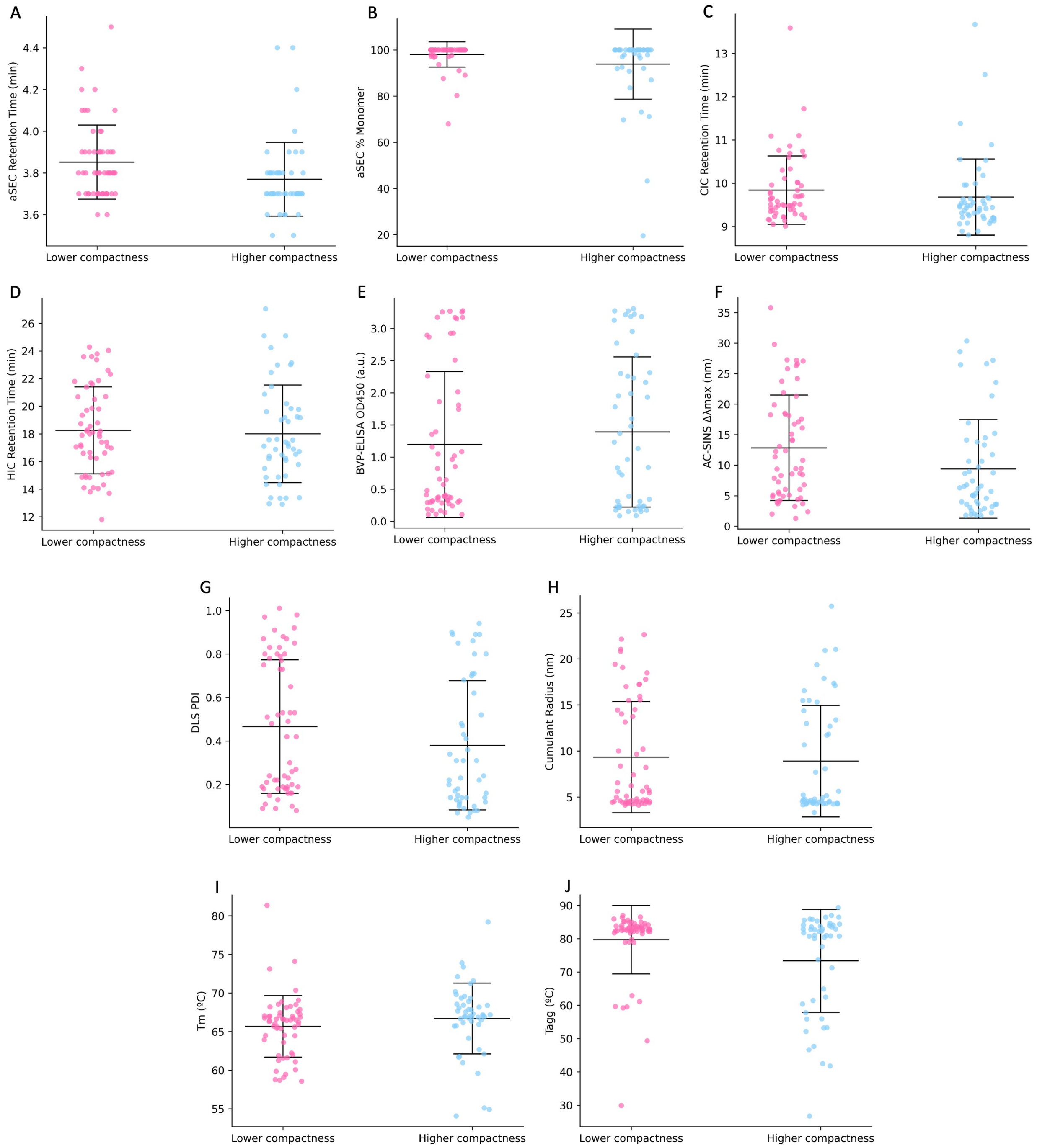

Figure S18. Results of experimental assays split into nanobodies with CDR3 loops of higher and lower compactness at a compactness score threshold of 1.25. (**A**) aSEC retention time (**B**) aSEC % monomer (**C**) CIC retention time (**D**) HIC retention time (**E**) BVP-ELISA OD450 (**F**) AC-SINS $\Delta\lambda$max (**G**) DLS PDI (**H**) Cumulant radius (**I**) Tm (melting temperature) (**J**) Tagg (aggregation temperature)

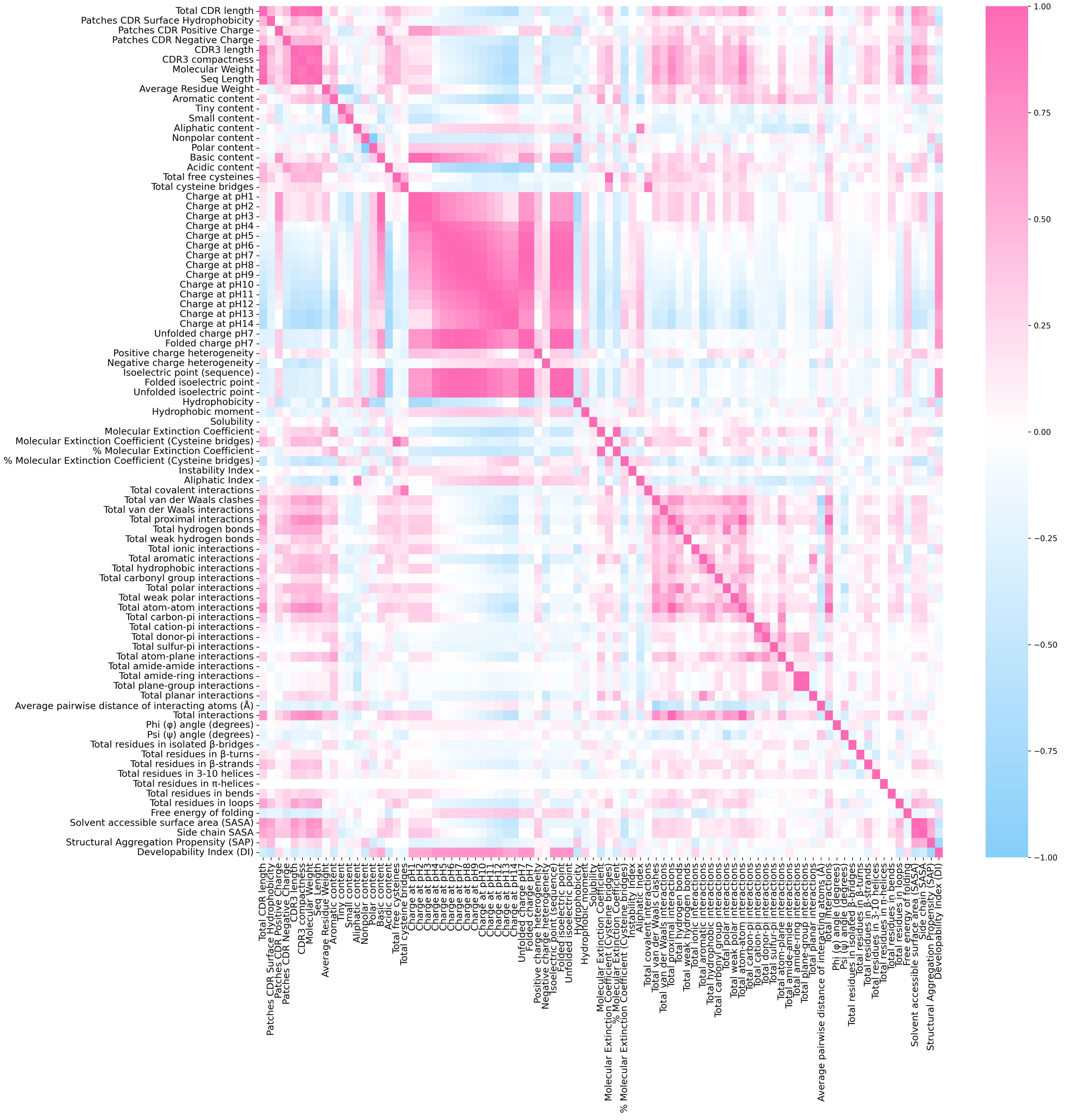

Figure S19. Heatmap of Spearman's rank correlations between all computational metrics calculated for the combined nanobody datasets only.

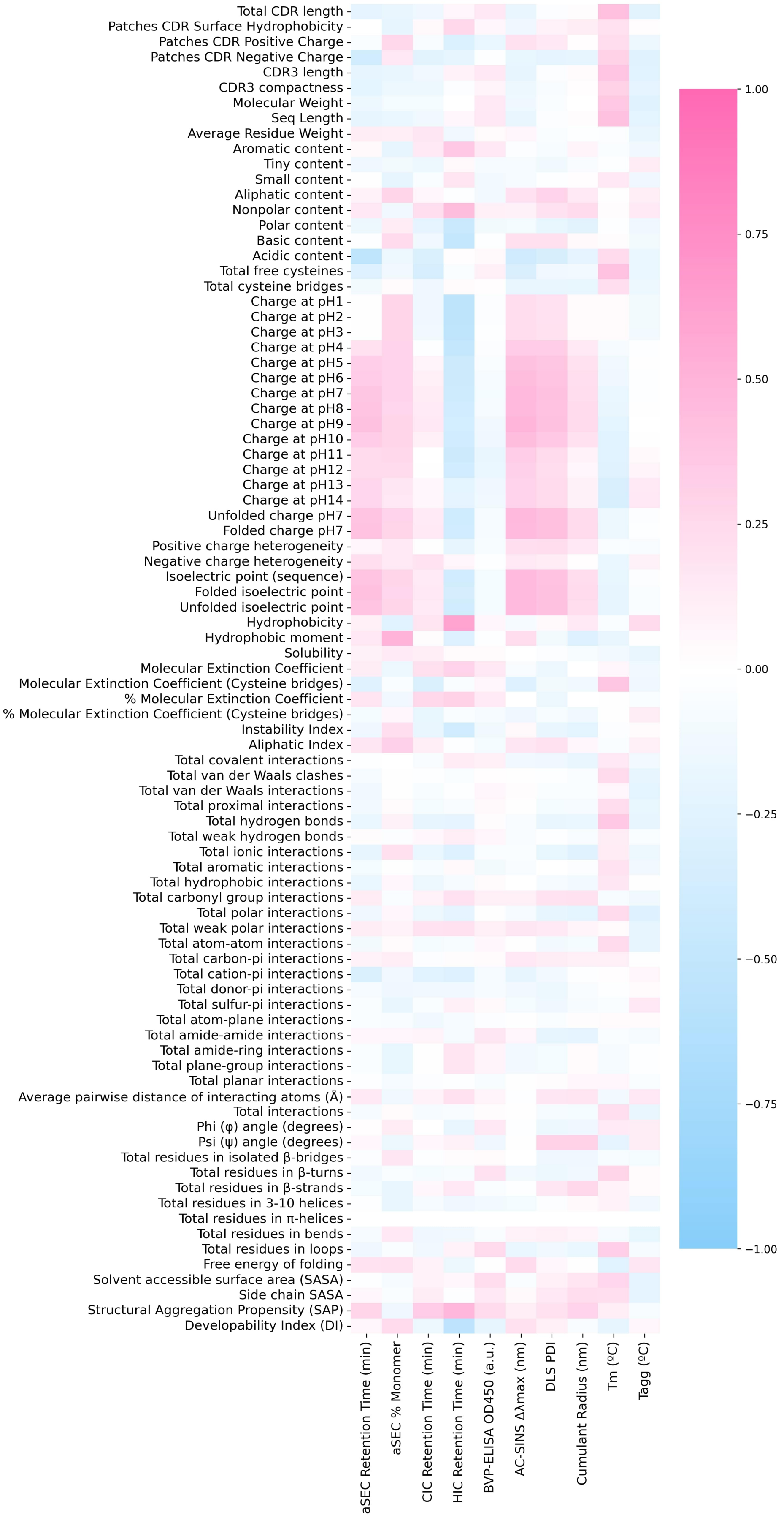

Figure S20. Heatmap of Spearman's rank correlations between all computational metrics calculated for the clinical-stage and proprietary nanobody datasets only against their *in vitro* assay results.

	Unbound PDB	Bound PDB		Unbound PDB	Bound PDB		Unbound PDB	Bound PDB
1	8f8v	8f8w	26	5tp3	5toj	51	7np9	7p2d
2	8f8v	8f8x	27	4krn	4kro	52	6dbg	6dbf
3	8gz6	8gz5	28	5h8d	5h8o	53	6dba	6dbg
4	7sah	6lr7	29	6y0e	6xzf	54	6dba	6dbf
5	7vpy	7vq0	30	3r0m	7ri2	55	6apq	4w2o
6	4b50	7aej	31	3r0m	3rjq	56	5wb1	4xt1
7	6u53	6u54	32	5sv4	5sv3	57	5wb2	4xt1
8	4w2p	4w2q	33	4s11	4s10	58	5hdo	5hgg
9	5vm4	5vm6	34	4s11	6h1f	59	5lz0	6ey0
10	7kgl	7kgk	35	4qgy	4y7m	60	6u50	6u51
11	7mfv	7kgk	36	4m3j	4m3k	61	6u50	6u52
12	7lvu	7lvw	37	5m2w	5mwn	62	7om5	7om4
13	5e7b	5e7f	38	5m2w	5m30	63	7omt	7omm
14	6fys	6fyw	39	7kkj	7kkl	64	2xa3	7r73
15	6ksn	7d5p	40	6ck8	6fyt	65	6cwk	6cwg
16	6ksn	7d5q	41	6z1z	6z20	66	6u14	6u12
17	3qxu	3qxt	42	5vlv	5vm0	67	6ru3	6ruv
18	7v0v	3k1k	43	3qxw	7rga	68	6ru3	7noz
19	7v0v	3ogo	44	3qxw	7rg7	69	2xxc	2xt1
20	1f2x	1g6v	45	3qxw	3qxv	70	2xxc	2xv6
21	6t2j	6sc5	46	5jmr	5jmo	71	2xxc	2xxm
22	6t2j	6sc9	47	6o89	6o8d	72	6jb9	1zvy
23	6t2j	6sc7	48	5vnv	5vnw	73	6jb9	6jb8
24	6t2j	6sc8	49	4dka	4dk6	74	5e03	5e5m
25	6t2j	6sc6	50	4dka	4dk3	75	5ivo	5ivn
						76	6apo	6app

Table S1. Pairs of PDB IDs for comparison of CDR3 compactness for bound and unbound nanobodies.

	Molecular				
1	Molecular Weight	29	Folded charge pH7	56	Total atom-atom interactions
2	Sequence Length	30	Positive charge heterogeneity	57	Total carbon-pi interactions
3	Average Residue Weight	31	Negative charge heterogeneity	58	Total cation-pi interactions
	Amino acid composition	32	Isoelectric point (sequence)	59	Total donor-pi interactions
4	Aromatic content	33	Folded isoelectric point	60	Total sulfur-pi interactions
5	Tiny content	34	Unfolded isoelectric point	61	Total atom-plane interactions
6	Small content	35	Hydrophobicity	62	Total amide-amide interactions
7	Aliphatic content	36	Hydrophobic moment	63	Total amide-ring interactions
8	Nonpolar content	37	Solubility	64	Total plane-group interactions
9	Polar content		Photochemical	65	Total planar interactions
10	Basic content	38	Molecular Extinction Coefficient	66	Average pairwise distance of interacting atoms (Å)
11	Acidic content	39	Molecular Extinction Coefficient (Cysteine bridges)	67	Total interactions
12	Total free cysteines	40	% Molecular Extinction Coefficient		Secondary structure
13	Total cysteine bridges	41	% Molecular Extinction Coefficient (Cysteine bridges)	68	Phi (ϕ) angle (degrees)
	Electrochemical		Stability	69	Psi (ψ) angle (degrees)
14	Charge at pH1	42	Instability Index	70	Total residues in isolated β-bridges
15	Charge at pH2	43	Aliphatic Index	71	Total residues in β-turns
16	Charge at pH3		Interactions	72	Total residues in β-strands
17	Charge at pH4	44	Total covalent interactions	73	Total residues in 3-10 helices
18	Charge at pH5	45	Total van der Waals clashes	74	Total residues in α-helices
19	Charge at pH6	46	Total van der Waals interactions	75	Total residues in bends
20	Charge at pH7	47	Total proximal interactions	76	Total residues in loops
21	Charge at pH8	48	Total hydrogen bonds		Thermodynamic
22	Charge at pH9	49	Total weak hydrogen bonds	77	Free energy of folding
23	Charge at pH10	50	Total ionic interactions		Solvent accessibility
24	Charge at pH11	51	Total aromatic interactions	78	Solvent accessible surface area (SASA)
25	Charge at pH12	52	Total hydrophobic interactions	79	Side chain SASA
26	Charge at pH13	53	Total carbonyl group interactions		Druggability
27	Charge at pH14	54	Total polar interactions	80	Structural Aggregation Propensity (SAP)
28	Unfolded charge pH7	55	Total weak polar interactions	81	Developability Index (DI)

Table S2. Additional *in silico* descriptors calculated for our nanobody datasets grouped by class according to Bashour *et al.* [3].

		CIC	HIC	BVP-ELISA	AC-SINS	DLS
KEY		Retention Time (min)	Retention Time (min)	OD_{450} (a.u.)	Binding (nm)	PDI
	Good	< 9	< 15	< 0.7	< 10	< 0.3
	Caution	9 - 10	15 - 18	0.7 - 1.3	10 - 25	0.3 - 0.6
	Poor	> 10	> 18	> 1.3	> 25	> 0.6

Table S3. Flag assignment and threshold values for *in vitro* assays.

Samples	CIC Retention Time (min)	HIC Retention Time (min)	BVP-ELISA OD450 (a.u.)	AC-SINS Δλmax (nm)	DLS PDI	Total CDR length	CDR3 length	CDR3 compactness	Patches CDR Surface Hydrophobicity	Patches CDR Positive Charge	Patches CDR Negative Charge
Brivekimig1_VHH1	9.25	13.35	1.95	26.45	0.41	33	16	1.35	81.36	0.26	0.22
Brivekimig1_VHH2	9.24	13.37	0.23	27.18	0.36	33	16	1.38	94.24	0.06	0.17
Brivekimig2_VHH1	9.38	16.60	3.16	7.25	0.09	24	8	0.83	106.99	0.00	0.00
Caplacizumab_VHH1	9.33	13.34	0.22	26.61	0.62	37	21	1.53	92.06	0.57	1.22
Enristomig_VHH1	9.23	15.23	0.11	4.38	0.26	20	5	0.56	79.36	0.05	0.00
Enristomig_VHH2	9.39	11.80	1.02	26.58	0.97	30	15	1.04	106.50	0.06	0.00
Envafolimab_VHH1	9.28	14.97	1.13	6.61	0.80	37	21	1.61	102.37	0.05	1.14
Erfonrilimab_VHH2	9.48	20.87	3.31	5.03	0.22	37	21	1.57	102.99	0.00	0.00
Gacovetug_VHH1	9.08	14.34	3.18	2.54	0.22	30	15	1.27	97.05	0.00	0.17
Gefurulimab_VHH1	9.67	15.50	0.24	28.59	0.89	32	17	1.42	97.04	0.06	0.18
Gefurulimab_VHH2	9.53	16.24	0.09	7.44	0.23	35	19	1.52	122.87	0.18	0.06
Gocatamig2_VHH1	9.70	18.74	0.29	9.43	0.73	26	11	0.82	138.68	0.00	1.11
Gontivimab_VHH1	9.37	19.24	1.93	6.15	0.17	35	19	1.36	99.40	0.04	0.11
Gontivimab_VHH2	9.37	19.18	0.72	5.71	0.14	35	19	1.36	91.42	0.04	0.11
Isecarosmab_VHH1	9.18	14.33	0.23	4.95	0.12	33	17	1.45	86.44	0.36	1.22
Isecarosmab_VHH2	9.51	17.81	0.33	6.03	0.16	24	8	0.80	96.49	0.00	0.00
Letolizumab_VHH1	9.49	16.97	1.39	9.48	0.87	27	11	1.00	95.13	0.27	0.28
Lofacimig_VHH1	9.31	18.90	3.27	2.92	0.10	39	23	1.49	155.47	0.00	0.00
Lofacimig_VHH2	8.89	19.84	2.30	1.79	0.31	35	20	1.32	119.68	0.00	1.53
Lunsekimig1_VHH1	10.63	17.06	3.25	27.24	0.51	32	16	1.23	73.40	0.00	0.00
Lunsekimig1_VHH2	9.96	16.88	3.23	15.21	0.10	35	19	1.39	116.89	0.00	0.00
Lunsekimig2_VHH1	9.20	18.28	0.24	3.97	0.18	26	11	0.85	116.19	0.00	0.05
Lunsekimig3_VHH1	8.89	13.38	0.17	2.04	0.14	36	18	1.48	97.22	0.10	1.88
Ozekibart_VHH1	9.44	15.78	0.17	14.49	0.86	32	16	1.29	79.67	0.21	0.00
Ozoralizumab_VHH1	9.69	14.04	0.27	29.78	0.08	24	8	0.98	83.65	0.10	0.00
Ozoralizumab_VHH2	9.65	17.21	0.19	5.09	0.21	24	8	0.85	111.02	0.00	0.00
Podentamig1_VHH1	9.31	15.12	3.28	4.21	0.53	28	14	0.92	86.31	0.02	0.00
Podentamig1_VHH2	9.43	17.09	0.11	18.5	0.13	24	8	0.83	98.59	0.00	0.00
Porustobart_VHH1	9.76	16.62	0.11	18.36	0.85	28	13	1.11	103.61	0.16	0.00
Produvofusp_VHH1	9.07	17.10	1.23	3.61	0.12	38	22	1.57	90.60	0.00	0.29
Rimteravimab_VHH1	9.06	16.9	0.15	3.99	0.14	34	18	1.43	89.51	0.00	0.65
Sonelokimab1_VHH1	9.41	17.11	0.09	11.74	0.71	32	16	1.32	104.10	0.07	0.24
Sonelokimab2_VHH1	9.55	16.38	0.83	3.23	0.05	31	17	1.35	97.97	0.23	1.45
Tarperprumig_VHH1	9.96	16.72	0.22	30.35	0.94	32	17	1.42	99.13	0.06	0.14
Tarperprumig_VHH2	9.22	14.86	0.17	3.68	0.24	28	13	0.89	99.60	0.30	0.14
Vobarilizumab_VHH1	9.48	17.09	3.17	35.79	0.42	30	15	1.01	85.49	1.19	0.62

Table S4. Flag assignment and raw values for *in vitro* and TNP metrics for the 36 clinical-stage sequences. The raw data is available at *github.com/oxpig/TNP*.

The constant region sequence used for the expression of Fc fusion constructs in an IgG1 format for *in vitro* assays:

GGGGSEPKSSDKTHTCPPCPAPELLGGPSVFLFPPKPKDTLMISRTPEVTCVVVDVSHEDPEVKFNWYVDGVEVHNAKTKPR
EEQYNSTYRVVSVLTVLHQDWLNGKEYKCKVSNKALPAPIEKTISKAKGQPREPQVYTLPPSREEMTKNQVSLTCLVKGFYPSD
IAVEWESNGQPENNYKTTPPVLDSDGSFFLYSKLTVDKSRWQQGNVFSCSVMHEALHNHYTQKSLSLSPG*
